# All-cause child mortality in minority and non-minority areas in Sichuan Province in Western China, 2008–2017

**DOI:** 10.1038/s41598-019-50616-z

**Published:** 2019-10-02

**Authors:** Zi-ling Zhao, Ming-hong Yao, Gang Zhang, Gong-hua Wu, Li Zhang, Ju-ying Zhang, Xiao Ma

**Affiliations:** 1 West China School of Public Health and West China Fourth Hospital, Sichuan University, Chengdu, Sichuan People’s Republic of China; 2 Sichuan Provincial Hospital for Women and Children, Affiliated Women and Children’s Hospital of Chengdu Medical College, Chengdu, Sichuan People’s Republic of China

**Keywords:** Paediatric research, Paediatric research, Epidemiology, Epidemiology

## Abstract

This study aimed to evaluate the disparity in the under-five mortality rate (U5MR) between minority and non-minority areas in Sichuan Province in Western China. Data for this study was obtained from the National Health Statistics Survey System. The Cochran-Armitage trend test was used to analyze the time trend of the U5MR. We conducted Poisson regression model to compare the differences of U5MRs between minority and non-minority areas. The U5MR in Sichuan province was reduced by 62.19% from 2008 to 2017, with the minority and non-minority areas reduced by 60.48% and 65.39%, respectively. The under-five mortality risk in minority areas was approximately 1.791 times (95% CI: 1.790–1.793; *P* < 0.01) that in non-minority areas. The primary cause of death of children under-five years old in minority areas was the respiratory disease, which was significantly higher than that in non-minority areas (*P* all < 0.01). The U5MR significantly declined both in minority and non-minority areas in Sichuan Province in Western China from 2008 to 2017. However, disparities still existed between minority and non-minority areas. Respiratory diseases were the main causes of death in minority areas and corresponding rates were higher than those in non-minority areas.

## Introduction

The health of children is largely dependent on their parents and the social and natural environment around them. The under-five mortality rate (U5MR), the neonatal mortality rate (NMR) and the infant mortality rate (IMR), which reflect the quality of health care services for children in a region, are generally used to measure a country or regional medical and health care levels and children’s overall health. Thus, reducing the U5MR is a long-term goal for all countries in the world^1^. For instance, the Millennium Development Goals (MDG) 4 points that the U5MR should be reduced by two-thirds between 1990 and 2015, and the latest Sustainable Development Goals (SDG) 3 states that all countries seek to reduce the NMR and U5MR to at least as low as 12 and 25 per 1,000 live births by 2030, respectively^2–4^. For a long time, many scholars have conducted numerous studies on the trends of the three indicators and their related factors and encouraged the government to formulate relevant measures to reduce mortality rates in children^5–7^. With the continuous development of the economy, a large amount of money is being used to improve children’s living conditions. U5MRs worldwide has decreased significantly over the past 20 years, but there are also great differences in child mortality around the world due to the unbalanced development of economic and health levels; the child mortality rates in developing countries are significantly higher than those in developed countries^8,9^.

Over the past few decades, China has made great strides in the economy, culture and health; the health of the people has continuously improved, and the U5MRs has declined significantly^10^. China is one of the few countries that has already completed the MDG 4 target, and the U5MRs in China declined from 50.8 deaths per 1,000 live births in 1996 to 10.7 deaths per 1,000 live births in 2015^11^. However, the number of children dying every year is still high compared to other developed countries due to the large population base; 181,500 children still died before their fifth birthday in 2015. At the same time, the U5MR in Western China is still high due to the difference in economic development and natural conditions, and it is especially concentrated in the minority areas of Western China^11^. The risk of death for children in minority areas and economically underdeveloped areas still needs attention^12,13^.

Sichuan is a multi-ethnic western province in China with the largest Yi nationality area, the only Qiang nationality area, and the second-largest Tibetan area. The area of minority areas accounts for over 60% of the Sichuan province’s area; however, most of the Sichuan minority areas are economically underdeveloped compared to other parts of Sichuan. The underdeveloped economy has always been an important factor restricting the improvement of children’s health^14^. For instance, Wu *et al*. found that the population in minority areas accounts for 11.5% of the total population of Sichuan, but the U5MRs in minority areas accounts for 20% of the total U5MRs in Sichuan^15^.

Therefore, the comparison and analysis of the trends and the cause of the U5MRs in minority and non-minority areas will provide specific measures for preventing the death of children in minority areas. At the same time, Sichuan’s success experience in reducing U5MR can be shared with other countries and regions with similar economic and health levels.

## Results

### Time trends in U5MR, NMR, and IMR

**Figure 1:**
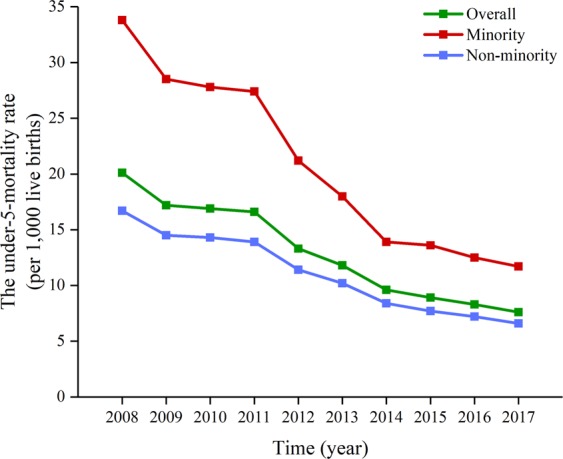
The time trend of U5MR in Sichuan Province.

**Figure 2:**
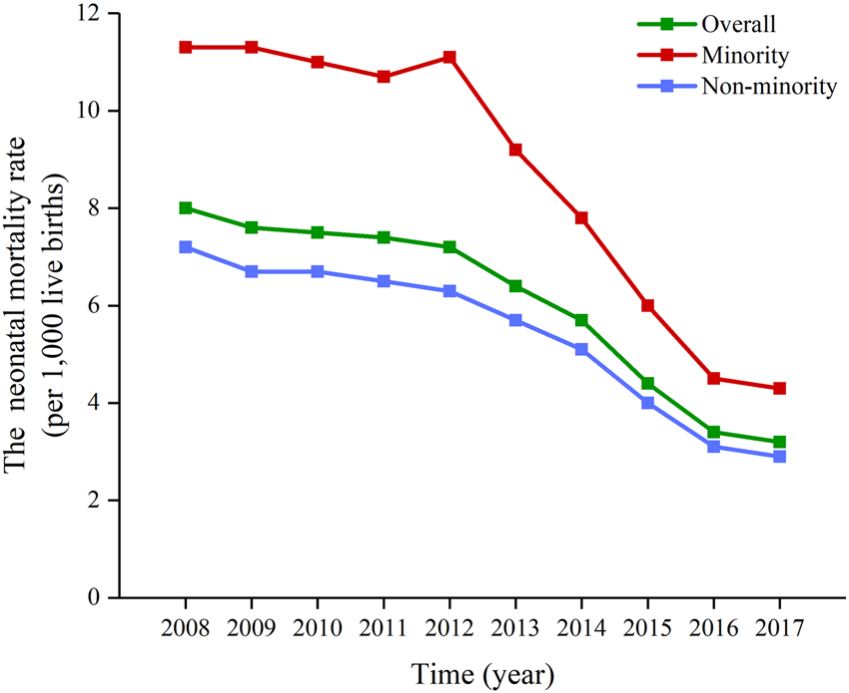
The time trend of NMR in Sichuan Province.

**Figure 3:**
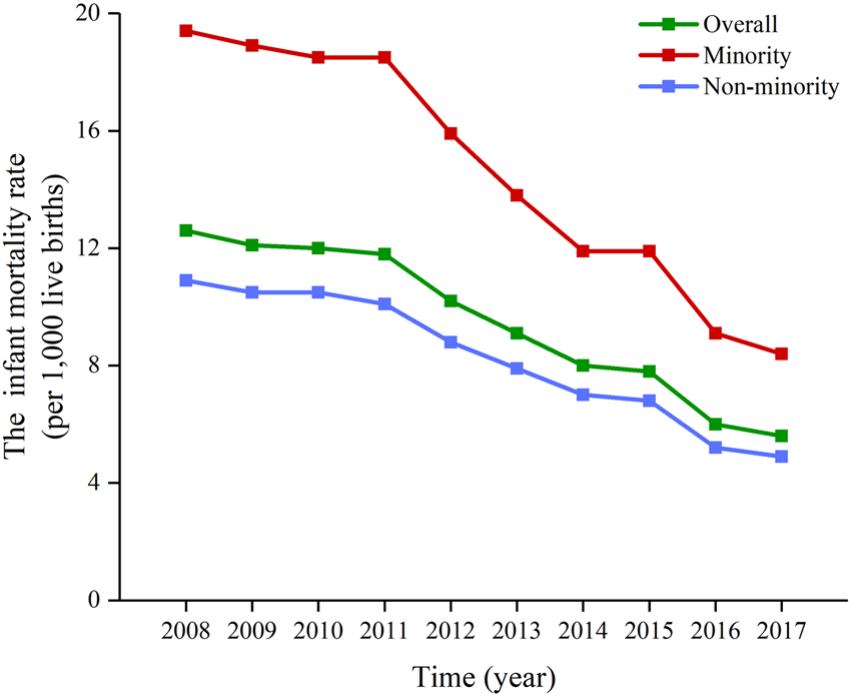
The time trend of IMR in Sichuan Province.

From 2008 to 2017, the overall U5MR in Sichuan Province decreased by 62.19% (χ^2^ = 9950.073, *p*_*trend*_ < 0.001), and the average annual decline rate was 10.24%. The U5MR also significantly decreased from 2008 to 2017 in both non-minority (decreased by 60.48%; χ^2^ = 6189.967, *p*_*trend*_ < 0.001) and minority (decreased by 65.39%; χ^2^ = 3939.284, *p*_*trend*_ < 0.001) areas; the average annual decline rate was 9.80% and 11.12%, respectively (Fig. 1). The time trend of NMR and IMR in Sichuan Province during 2008–2017 is shown in Figs 2 and 3, respectively. The overall NMR and IMR in Sichuan Province was decreased by 60.00% (χ^2^ = 3498.352, *p*_*trend*_ < 0.001) and 55.56% (χ^2^ = 4765.584, *p*_*trend*_ < 0.001), respectively. And the average annual decline rate for NMR and IMR in Sichuan province was 9.68% and 8.62%, respectively. The NMR and IMR also all significantly decreased from 2008 to 2017 in both non-minority (NMR: decreased by 59.72%; χ^2^ = 2383.316, *p*_*trend*_ < 0.001; IMR: decreased by 55.05%; χ^2^ = 3268.949, *p*_*trend*_ < 0.001) and minority (NMR: decreased by 61.95%; χ^2^ = 1161.143, *p*_*trend*_ < 0.001; IMR: decreased by 56.70%; χ^2^ = 1565.316, *p*_*trend*_ < 0.001) areas.

### Comparison of the U5MR differences between minority and non-minority areas

After adjusting for age, gender and time, the under-five mortality risk in minority areas was approximately 1.791 times (95% CI: 1.790–1.793; *P* < 0.01) that in non-minority areas (Table 1).

**Table 1:** Comparison of the U5MR between in minority and non-minority areas.

Variable	β(95%CI)	SE	RR (95%CI)	Waldχ^2^	P
Region	0.583 (0.582, 0.584)	0.003	1.791 (1.790, 1.793)	35527.43	<0.01
Age	0.393(0.389, 0.397)	0.002	1.481 (1.476, 1.487)	42733.86	<0.01
Year	−0.106 (−0.108, −0.104)	0.001	0.899 (0.898, 0.901)	40063.26	<0.01
Sex	0.613(0.607, 0.619)	0.003	1.846 (1.835, 1.857)	1951.09	<0.01

### Age composition of deceased children under-five years old

**Figure 4:**
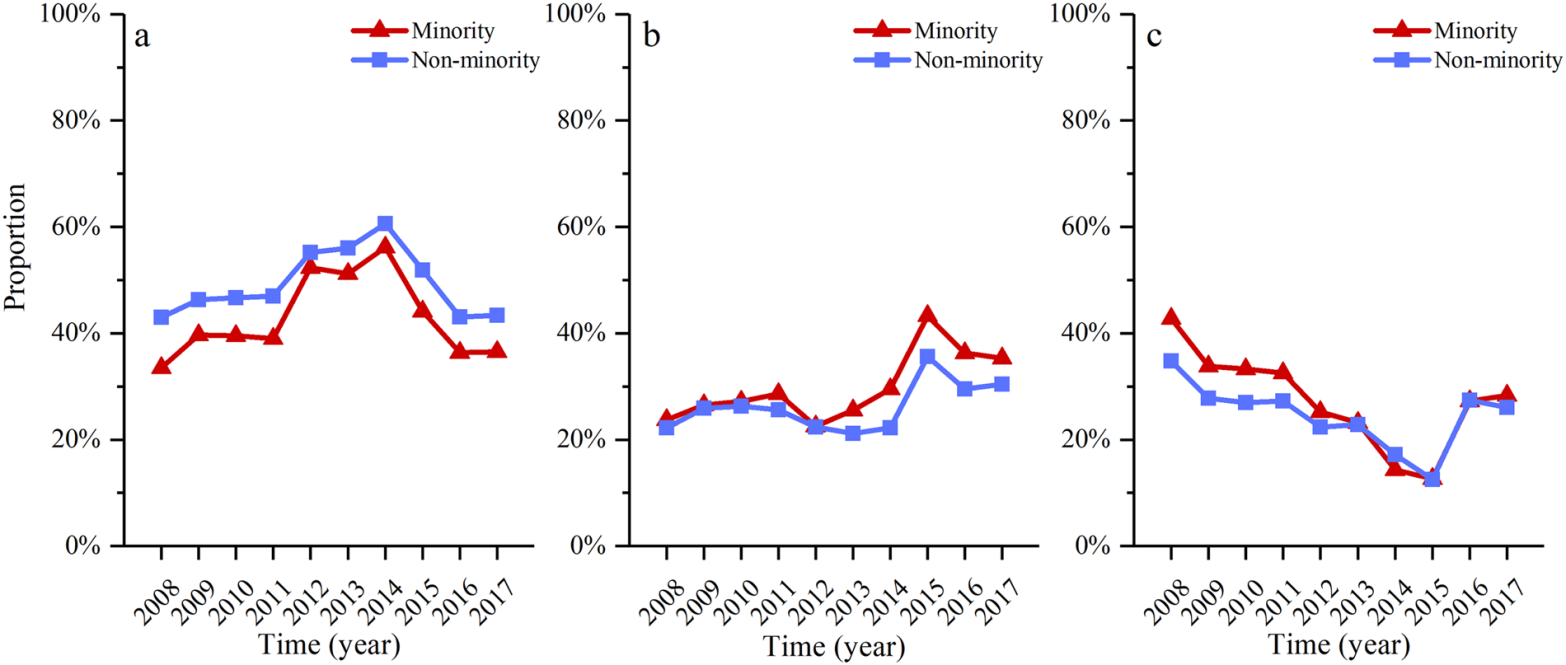
Age composition of deceased children under-five years old from 2008 to 2017: (**a**) Neonatal, (**b**) 29d-12 month, (**c**) 13–59 months.

Figure 4 shows the age composition of deceased children under-five years old from 2008 to 2017 in both minority and non-minority areas. There was no significant change in proportion of deceased neonates in either minority (33.5% vs 36.5%) or non-minority (43.0% vs 43.4%) areas. The proportion of the deceased children aged 29 days-12 months increased from 2008 to 2017 in both minority (23.7% vs 35.3%) and non-minority (22.2% vs 30.4%) areas, but the proportion of the deceased children aged 13–59 months decreased from 2008 to 2017 in both minority (42.8% vs 28.3%) and non-minority (34.8% vs 26.1%) areas.

### The U5MR causes between minority and non-minority areas

**Figure 5:**
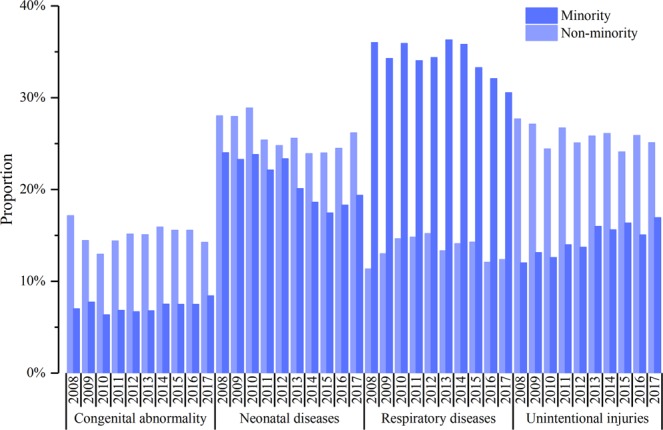
The top four causes of death of children under-five years old in Sichuan Province.

From 2008 to 2017, the main leading causes of death of children under-five years old in Sichuan province were respiratory diseases, neonatal diseases, unintentional injuries and congenital abnormalities (Fig. 5). The primary cause of death of children under-five years old in minority areas was respiratory diseases, which was significantly higher than that in non-minority areas (*P* all < 0.01). The proportion of children whose death could have been prevented in minority areas (from 59.8% in 2008 to 50.1% in 2017) was significantly higher than that in non-minority areas (from 45.3% in 2008 to 39.6% in 2017) (*P* all < 0.01).

### Analysis of the utilization of health care services of *deceased* children under-five years old

**Figure 6:**
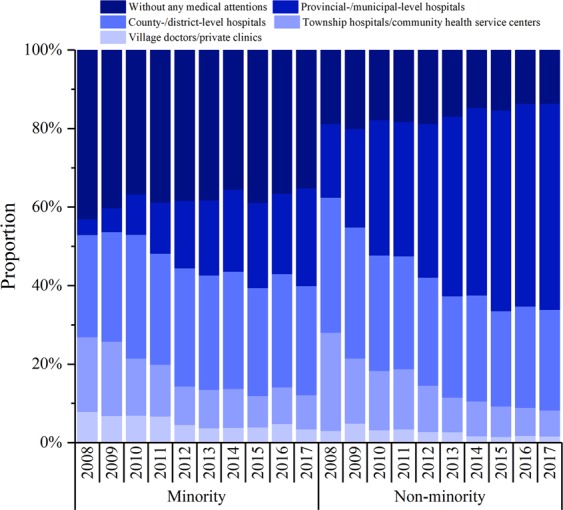
Health care services for children under-five in Sichuan Province of China.

Figure 6 shows the proportions of children who sought medical services before death from 2008 to 2017 in both minority and non-minority areas. The overall proportion of children who sought medical services increased from 66.5% in 2008 to 80.7% in 2017, but the proportion of children who sought medical services in minority areas (from 57.0% in 2008 to 64.9% in 2017) was significantly lower than that in non-minority areas (from 81.3% in 2008 to 86.5% in 2017) (*P* all < 0.01).

## Discussion

This study analyzed the time trends of U5MR, NMR and IMR in Sichuan province from 2008 to 2017. From 2008 to 2017, the U5MR decreased from 20.1 in 2008 to 7.6 in 2017 per 1,000 live births in Sichuan province, from 33.8 in 2008 to 11.7 in 2017 per 1,000 live births in minority areas, from 16.7 in 2008 to 6.6 in 2017 per 1,000 live births in non-minority areas. For the NMR and IMR, the overall NMR and IMR in Sichuan province was decreased by 60.00% and 55.56%, respectively. The NMR and IMR also all significantly decreased from 2008 to 2017 in both non-minority and minority areas. Although the U5MR in Sichuan province significantly decreased from 2008 to 2017, it is still higher than in developed countries of the world and the national level. For instance, the GBD study 2017 shows that the U5MRs in the United States, the United Kingdom, France, Australia, Germany, Russia and China are 133.51, 90.00, 75.26, 80.63, 76.54, 129.81, and 205.23 per 100,000 live births, respectively (https://vizhub.healthdata.org/gbd-compare/). This also suggests that more efforts are needed to reduce the U5MR in Sichuan Province.

The disparities in U5MR, NMR and IMR between minority and non-minority areas in Sichuan province narrowed over time, however, the gap among Sichuan province’s minority and non-minority areas still exists. In recent years, the overall economy and health level in Sichuan have been continuously improved, and the government has invested substantial funds to strengthen the construction and development of medical and health care undertakings in minority and non-minority areas, which could be an important contributor to the reduction in U5MR, NMR and IMR in minority areas^10,16^. There are several underlying reasons could explain the differences between minority and non-minority areas. First, differences in economic levels between minority and non-minority areas can affect child mortality rate. Xia *et al*. have confirmed a significant negative relationship between per capita GDP and U5MR in four western provinces of China^14^. The economic level of minority areas in Sichuan is much lower than that of non-minority areas (http://tjj.sc.gov.cn/tjcbw/tjnj/2018/zk/indexch.htm). At the same time, the minority areas experience financial difficulties, limited capacity for their own health systems, and insufficient government investment^17^. Second, the uneven distribution of health care resources in between minority and non-minority areas is important reason for the U5MR, NMR and IMR being higher in minority areas than those in non-minority areas^18,19^. The health service in minority areas is poor, the township hospitals are insufficient, the equipment is lacking, the technical force of the health technicians is weak, and the medical service capacity is insufficient^20^. Ren *et al*. found that the gap in qualified doctors between minority and non-minority counties is increasing, which may be a reason for the higher U5MR in minority areas^16^. Third, poor of accessibility to health care is another important reason. He *et al*. and Kang *et al*. have demonstrated that the proportion of deceased children under-five years old who used health services in rural areas was significantly lower in urban areas^21,22^. Most minority counties in Sichuan Province are located in rural areas, and traffic conditions and medical services are worse than non-minority areas, reducing children’s access to health care services. Therefore, the governments at all levels should further improve the allocation of health resources in minority areas, increase the total amount of health inputs in minority areas, optimize investment planning, cultivate health human resources, and increase the service capacity of the medical and health institutions in minority areas.

The proportion of neonatal deaths to the total deaths of children under-five years old was higher in minority areas than that in non-minority areas. At the same time, approximately half of the deceased children were not treated before death in minority areas; the proportion of children who sought medical services at district/county or higher-level hospitals is found to be significantly lower in minority areas than that in the non-minority areas. The possible reasons are as follows: first, the children living close to a healthcare facility might be more likely to obtain medical services before the disease progresses^23^. However, the minority areas are sparsely populated, the natural environment is poor, most residents live far away from medical services, and the accessibility of medical services is poor. Second, the quality of medical services also affects the outcome of medical services^24^ and there is a negative relationship between the number of hospitals per 100 thousand inhabitants and IMR^25^. But the number of medical service providers in minority areas is small, the educational level of doctors is relatively lower, and the hospital equipment is very basic. Third, the hospital delivery rate is an important factor affecting infant mortality, especially neonatal mortality, which is related to prenatal health care, medical service capacity and residents’ traditional habits^26^. The education level of people in minority areas is lower, many pregnant women are reluctant to go to the hospital for childbirth, and the hospital delivery rate in minority areas is generally lower.

There are differences in the causes of death among children under-five years old in minority and non-minority areas. The death of children under-five in minority areas is mainly due to respiratory diseases, with pneumonia as the main component of respiratory disease, which is similar to that in other low-income regions^27^. Meanwhile, the proportion of unintentional injuries and neonatal diseases in minority and non-minority areas are also relatively high. It also suggests that we need to continue to promote children’s safety education and maternal health. Avoidable death means that with effective medical intervention, the death could have been avoided^28^. This metric has been used in many countries to evaluate the progress of health care and evaluate the effectiveness of health services. This study shows that the proportion of children who died from preventable causes in minority areas was significantly higher than that in non-minority areas. Through the above analysis, the government should establish and improve the maternal and child health care network and perform specialized training and retraining in minority areas. Incorporating pneumonia vaccines into the free immunization program is another method to prevent and control the mortality caused by infectious disease and reduce the risk of death in minority areas.

## Limitations

This study has some limitations. First, the U5MRs, NMR and IMR data were collected from the National Health Statistics Survey System in Sichuan, which might have an under-reporting bias. Second, although we have taken measures to improve the accuracy of diagnosis and the quality of data reports, there may be inaccuracies in the diagnoses of cause of death in children, especially in minority areas^21^. Third, some basic information, such as household income, the educational level of the parents, the exposure to risk factors associated with pregnant women, and the distance from a home to a medical institution, was not collected, which prohibited an analysis of associations with these factors.

## Methods

### Data source

There are 67 and 116 minority counties and non-minority counties in Sichuan Province, respectively. The list of minority counties can be obtained from the website of the Ethnic and Religious Affairs Commission of Sichuan Province (http://www.scmzw.gov.cn/zwgk/tzgg/201409/t20140926_1427.html). The methods to calculate the U5MR, NMR, and IMR are reported in previous studies^29^. The U5MR, NMR and IMR were adjusted based on the 2010 Population Census. Information on causes of death was collected from the Maternal and Child Health Surveillance System and grouped into 14 categories (Supplementary Table S1); these categories were mapped to aggregated codes of the International Classification of Diseases 10th version (ICD-10). The avoidable deaths of children under 5 years of age included diarrhoea, septicaemia, meningitis, pneumonia, neonatal tetanus, drowning, accidental asphyxia, accidental poisoning, accidental fall and birth asphyxia^30^. Data for this study was obtained from the National Health Statistics Survey System. The details regarding the data collection, the quality control process, and the ascertainment cause of death have been reported in previous studies^11,21,31^.

### Statistical analysis

The time trends of the U5MR was analysed by the Cochran-Armitage trend test. Comparisons of the U5MRs between in minority and non-minority areas were explored using the Poisson regression model after adjusting for age, sex, and time. The Chi-Square Test was used for comparing the age composition of deceased children under-five years old. All analyses were performed using the SPSS statistical package (SPSS, Inc. Chicago, IL, USA). A *P* value < 0.05 was used to define the level of significance.

### Ethics approval and consent to participate

The data used in this study were derived from the National Health Statistics Survey System, which is derived from the official report cards approved by the government in China. The medical institutions, towns and communities of our country have legal obligations to submit the results of children’s deaths to the Provincial Maternal and Child Health Hospital. There is no longer need separate ethical approval and personal informed consent. The author, Ms. Zhao, as the officer of the Provincial Maternal and Child Health Hospital of Sichuan, was authorized by the Provincial Health Planning Commission to undertake the organization, implementation, information collection and analysis of the survey. The government publishes the results of child deaths on official websites every year at the same time. Our research focused on the aggregate level of minority and non-minority areas rather than individual populations; therefore, individual consent to participate was dispensable.

## Conclusions

The U5MRs significantly declined in the minority and non-minority areas in Sichuan Province of Western China from 2008 to 2017. However, disparities still exist between minority and non-minority areas. Respiratory diseases were the main causes of death in minority areas and were more prevalent than those in non-minority areas. The proportion of children who received treatment before death in non-minority areas was lower than that in minority areas. The proportion of preventable deaths in children in minority areas was higher than that in non-minority areas.

## Supplementary information

Table S1. Causes of deaths categorization and mapping of the International Classification of Diseases, Revision 10.
