# Supplementary material for: All-cause child mortality in minority and non-minority areas in Sichuan Province in Western China, 2008–2017
Source: Sci Rep. 2019 Oct 2;9:14154. doi: 10.1038/s41598-019-50616-z (PMC6775076; doi:10.1038/s41598-019-50616-z)
Supplement: Supplementary file 1 — Table S1. Causes of deaths categorization and mapping of the International Classification of Diseases, Revision 10. [file 41598_2019_50616_MOESM1_ESM.docx]

*All-cause child mortality in minority and non-minority areas in Sichuan Province in Western China, 2008–2017*

Zi-ling Zhao^1,2^, Ming-hong Yao^1^,Gang Zhang^2^, Gong-hua Wu^1^, Li Zhang^1^, Ju-ying Zhang^1*^, Xiao Ma^1*^

^1^ West China School of Public Health and West China Fourth Hospital, Sichuan University, Chengdu, Sichuan, People’s Republic of China

^2^ Sichuan Provincial Maternal and Child Health Hospital, Chengdu, Sichuan, People’s Republic of China

E-Mails: [344399976@qq.com (Zi-ling Zhao](mailto:344399976@qq.com%20(Zi-ling%20Zhao%20) ); ymhldjxa@sina.com (Ming-hong Yao);

[1419174430@qq.com](mailto:1419174430@qq.com) (Gang Zhang);[904439105@qq.com](mailto:904439105@qq.com) (Gong-hua Wu);

[1520365941@qq.com](mailto:1520365941@qq.com) ( Li Zhang);[1643160963@qq.com](mailto:1643160963@qq.com) (Fang Liao); 2465297828@qq.com( Hua-yan Quan); 624100732 @qq.com (Qian Xiao);

juying@163.com( Ju-ying Zhang); antiaids@163.com(Xiao Ma)

^*^Author to whom correspondence should be addressed; E-Mail: antiaids@163.com(Xiao Ma); juying@163.com( Ju-ying Zhang).

Table S1. Causes of deaths categorization and mapping of the International Classification of Diseases, Revision 10.

| Disease categories | ICD10 |
| --- | --- |
| Respiratory diseases | H65-H66, J00-J22, J30-J98, P23-P24, |
| Neonatal diseases | A33, P01.0-P02.9, P03-P22, P24-P96(excluding P78.3) |
| Unintentional injuries | V01-Y89 |
| Congenital abnormality | Q00-Q99 |
| Digestive system disease | A00-A09, P78.3, K00-93 |
| Other conditions | N70-N73, O00-O99, U04, F01-F99, H00-H61, H68-H93, L00-L98, M00-M99 |
| Infectious and parasitic diseases | A15-A32, A36-A99, B00-B99, P36,G03,G04 |
| Nervous system disease | A39.0, G00, G06-G98 |
| Tumor | C00-D48 |
| Hematological and hematopoietic diseases | D50-D53, D55-D89 (excluding D64.9) |
| Circulatory disease | I00- I99 |
| Endocrine, nutritional and metabolic diseases | E00-E34, E40-E88 |
| Urinary System Diseases | N00-N28, N30-N32, N34-N64(excludingN38.0), N75-N98 |
